# Neurofeedback of the difference in activation of the anterior cingulate cortex and posterior insular cortex: two functionally connected areas in the processing of pain

**DOI:** 10.3389/fnbeh.2014.00357

**Published:** 2014-10-15

**Authors:** Mariela Rance, Michaela Ruttorf, Frauke Nees, Lothar R. Schad, Herta Flor

**Affiliations:** ^1^Department of Cognitive and Clinical Neuroscience, Medical Faculty Mannheim, Central Institute of Mental Health, Heidelberg UniversityMannheim, Germany; ^2^Computer Assisted Clinical Medicine, Medical Faculty Mannheim, Heidelberg UniversityMannheim, Germany

**Keywords:** real-time fMRI, pain, anterior cingulate cortex, posterior insula, neuromodulation, connectivity

## Abstract

The aim of this study was the analysis of the effect of a learned increase in the dissociation between the rostral anterior cingulate cortex (rACC) and the left posterior insula (pInsL) on pain intensity and unpleasantness and the contribution of each region to the effect, exploring the possibility to influence the perception of pain with neurofeedback methods. We trained ten healthy subjects to increase the difference in the blood oxygenation level-dependent response between the rACC and pInsL to painful electric stimuli. Subjects learned to increase the dissociation with either the rACC (state 1) or the pInsL (state 2) being higher. For feedback we subtracted the signal of one region from the other and provided feedback in four conditions with six trials each yielding two different states: [rACC—pInsL increase (state 1), rACC—pInsL decrease (state 2), pInsL—rACC increase (state 2), pInsL—rACC decrease (state 1)]. Significant changes in the dissociation from trial one to six were seen in all conditions. There were significant changes from trial one to six in the pInsL in three of the four conditions, the rACC showed no significant change. Pain intensity or unpleasantness ratings were unrelated to the dissociation between the regions and the activation in each region. Learning success in the conditions did not significantly correlate and there was no significant correlation between the two respective conditions of one state, i.e., learning to achieve a specific state is not a stable ability. The pInsL seems to be the driving force behind changes in the learned dissociation between the regions. Despite successful differential modulation of activation in areas responsive to the painful stimulus, no corresponding changes in the perception of pain intensity or unpleasantness emerged. Learning to induce different states of dissociation between the areas is not a stable ability since success did not correlate overall or between two conditions of the the same state.

## Introduction

The number of studies utilizing neuromodulation to alter behavior, cognition, and emotional processing has been growing in the past ten years. Applications include the modulation of the blood oxygenation level-dependent (BOLD) response in single brain regions such as altering motor function via modulation of the ventral or dorsal premotor cortex (Sitaram et al., 2012; Zhao et al., 2013), processing of emotional visual cues by modulating the anterior insula (Caria et al., 2010), craving in smokers by modulating the anterior cingulate cortex (Li et al., 2013), or improving working memory performance by modulating the dorsal lateral prefrontal cortex (Zhang et al., 2013). While in some studies altered brain activation was associated with changes in behavior, other studies showed that self-modulation of brain activation is possible in the absence of behavioral effects (Weiskopf et al., 2003; Johnston et al., 2011; McCaig et al., 2011; Birbaumer et al., 2013). More recently, real-time functional magnetic resonance imaging (rt-fMRI) has not only been used in the modulation of specific regions of the brain but of active networks defined by connectivity analyses or real-time pattern classification (Esposito et al., 2003; Laconte et al., 2007; Sitaram et al., 2011; Berman et al., 2013; Koush et al., 2013; Ruiz et al., 2013; Zotev et al., 2013; Scharnowski et al., 2014; Zilverstand et al., 2014). In the light of these results an important but so far under-investigated issue is the local specificity of neuromodulation of single regions, differential effects of up- or downregulation of BOLD activation and their influence on the active network and behavior.

Pain is a complex sensory and emotional experience and its alteration by rt-fMRI might be a challenge. It has been shown that pain perception involves a distributed network (Apkarian et al., 2005; Brodersen et al., 2012; Wager et al., 2013) that is, moreover, also involved in other functions (Iannetti and Mouraux, 2010; Legrain et al., 2011; Cauda et al., 2012b). Moreover, pain perception is subject of many modulating factors such as cognitive, emotional, and learning processes (Bingel et al., 2006; Diesch and Flor, 2007) that involve other brain circuits (Villemure and Bushnell, 2002, 2009; Wiech et al., 2006). Decharms et al. (2005) trained healthy controls while they received painful thermal stimulation to decrease and increase the BOLD signal in the rACC. The magnitude of the change in the activation was associated with the magnitude of the change in the ratings. The authors also transferred their design to chronic pain patients who subsequently reported pain relief. However, a replication of these results is still missing (Chapin et al., 2012).

We (Rance et al., 2014) previously examined and compared the effect of separately up- and downregulating two regions that are part of the pain processing network: the rACC, involved in the tonic aversive state elicited by pain (Apkarian et al., 2005; Qu et al., 2011) and the left posterior insula (pInsL), a region that processes the sensory aspect of pain perception (Rainville et al., 1997; Frot et al., 2007). In this study successful up- and downregulation of the rACC and pInsL was trained. The subjects acquired successful pInsL up- and downregulation and successful rACC down- but not upregulation. Successful modulation was not accompanied by a change in perceived pain intensity or unpleasantness. Modulation of one region also affected the second region, implying that the network seen active in the presence of the painful electrical stimulus was affected. Better learning was associated with higher dissociation between the two regions. Moreover, higher dissociation during upregulation of the pInsL correlated positively with an increase in pain unpleasantness ratings. These results suggest that the state of the pain-related network plays a role in the learning of self-modulation of the activation of single nodes.

In the light of these findings the current study aimed at examining and comparing the effects of a combined difference feedback of the two regions, rACC and pInsL, thus permitting to analyze the effect of the disruption of a part of the pain processing network. Similar to our previous study ten healthy subjects trained on four consecutive days. Two distinct states were trained: activation of the rACC higher than pInsL activation (state 1) or activation of pInsL higher than rACC activation (state 2). The goal of the training was to increase the difference. Each state was practiced in two distinct conditions; state 1A: rACC—pInsL increase (arrow up), state 1B: pInsL—rACC decrease (arrow down), state 2A: pInsL—rACC increase (arrow up), state 2B rACC—pInsL decrease (arrow down). For a detailed list of the balancing protocol see Supplementary Table SI. We assessed pain intensity and unpleasantness ratings after each training trial and recorded the strategy used. In the previous study we saw no differential contribution of the modulation of the two regions on the perception of pain intensity and unpleasantness. Based on our previous findings, we expected to find an effect of dissociating two functionally connected regions, which are part of a wider network active in the presence of painful stimulation, on pain intensity and unpleasantness ratings. We examined differences in the contributions of the two regions to the successful modulation of both. We thus compared within each condition (state) changes in the single regions of those subjects who were successful with those who did not learn the modulation of the combined regions.

## Materials and methods

### Participants

Ten healthy adults with a mean age of 27.8 years (*SD* = 4.71, range: 22–35), six females (*M* = 29.0, *SD* = 5.25) and four males (*M* = 26.0, *SD* = 3.65), were examined. All subjects were right-handed as assessed by the Edinburgh Handedness Inventory (Oldfield, 1971) and recruited by announcements at the local university. We defined cardiovascular or neurological disorders, brain injury, current or chronic pain, current analgesic medication, pregnancy, lifetime and current substance abuse or dependence, any mental disorder, and metallic implants as exclusion criteria. The study adhered to the Declaration of Helsinki and was approved by the Ethics Committee of the Medical Faculty Mannheim, Heidelberg University, Germany. All subjects gave written informed consent after a detailed description of the complete study and received a reimbursement of 60 €.

### Stimulation protocol

Painful monopolar transcutaneous electric stimuli (Digitimer, DS7A, Welwyn Garden City, UK) were applied to the base of the right fourth digit using a concentric stainless steel bipolar needle electrode (Nihon Kohden, Tokyo, Japan), which permitted the stimulation of Aδ nociceptive fibers located in the epidermis (Inui et al., 2002; Yoshino et al., 2010). Pulses lasted 1 ms and were applied with a frequency of 2 Hz. The method of limits was used to determine individual detection and pain thresholds, averaging over the last 2 of 3 ascending and descending stimulation sequences. Stimulation strength was set at 70% between pain threshold and pain tolerance and adjusted to be rated between 6 and 7 on an 11 point verbal rating scale (ranging from 0 = no pain to 10 = strongest imaginable pain). The individually adjusted mean stimulation current was 1.97 mA (*SD* = 1.51), the pre-baseline intensity of this stimulus was rated as 6.30 (*SD* = 1.06) and the unpleasantness as 5.80 (*SD* = 1.23). The post-baseline stimulus intensity was rated 6.10 (*SD* = 2.44) and the pain unpleasantness 6.50 (*SD* = 2.69) and served as reference for the ratings of the following training trials.

### rt-fMRI feedback protocol

**Figure 1:**
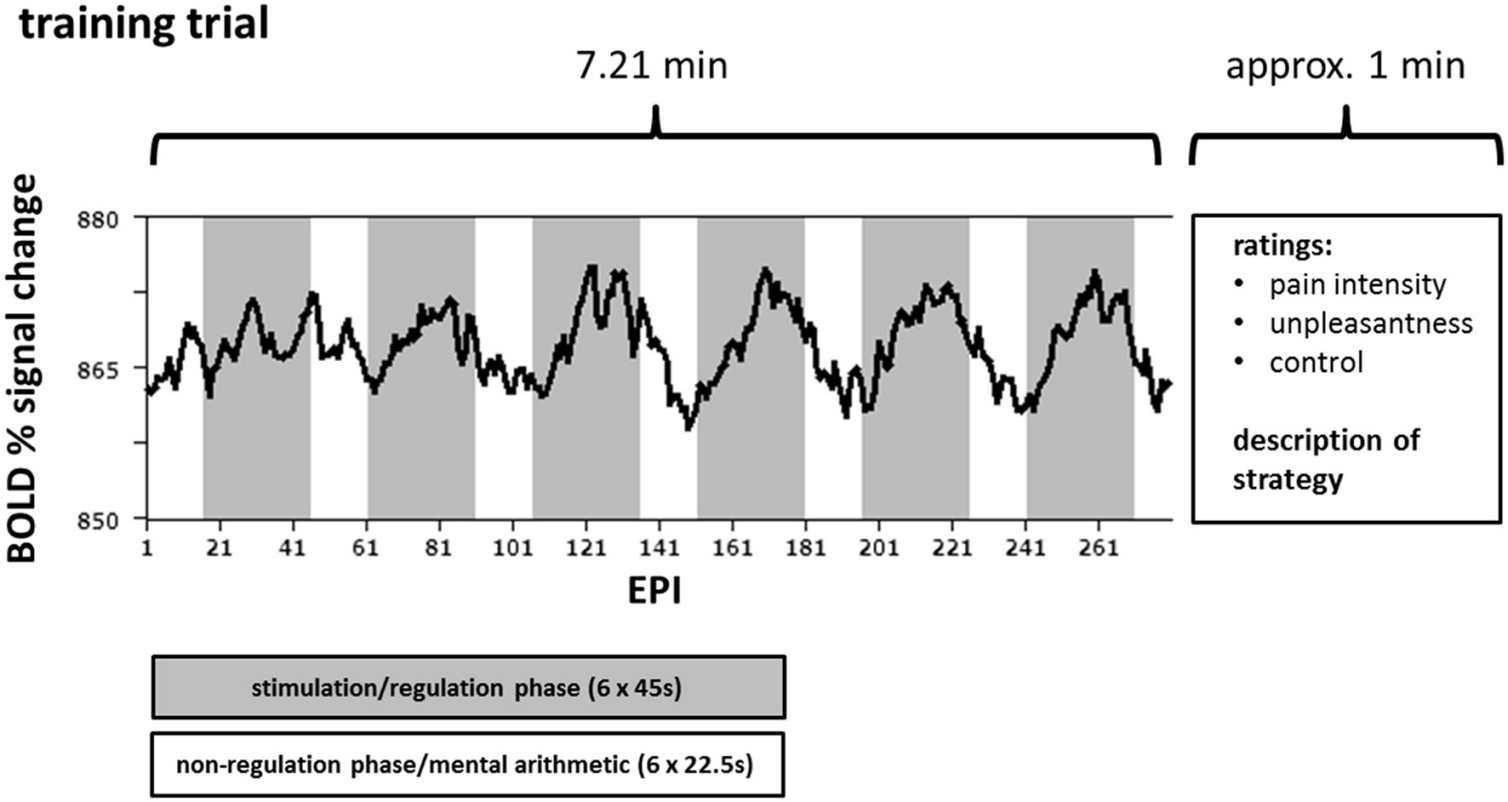
**Course of one training trial for one target region**. Over the course of one training trial (290 echo planar imaging (EPI) sequences) the participants attempted either up- or downregulation of the difference in the activation of the rostral anterior cingulate cortex (rACC) and the left posterior insula (pInsL) during 6 regulation phases each lasting 45 s (gray) while receiving painful electrical stimulation. Between the regulation phases, i.e., in the non-regulation phases (white), each lasting 22.5 s, the subjects performed mental arithmetic. An exemplary blood oxygenation level-dependent (BOLD) time course of one target area is superimposed in black.

The neurofeedback protocol was identical to the one described in detail in our previous study (Rance et al., 2014): a baseline run accompanied by an anatomical scan on one day followed by 24 training trials spread over the course of four consecutive days. Each training day consisted of six successive training trials; each trial was composed of six regulation phases (45.0 s) and six non-regulation phases (22.5 s) evenly distributed across each session. During the regulation phases, painful electric stimuli were applied along with the real-time feedback (Figure 1). The feedback screen consisted of a moving blue or yellow ball on a black background accompanied by a stationary white arrow indicating the vertical direction in which the ball should be moved. Ball movements were calculated from changes in the computed BOLD signal of the regions (rACC and pInsL). The positions of the regions of interest (ROIs) were determined online and monitored throughout the trials. The criteria for the positions of the ROIs were a) lying in the most significant cluster active during the regulation phase and b) being in the respective areas in the rACC and pInsL. If two foci of online activation in the rACC and pInsL were present, the location of the ROIs of the baseline run was used to guide the positioning. The feedback was updated every 1.5 s according to the repetition time of the fMRI protocol and calculated as the difference of the percent signal change in the two ROIs. The ball color was similar, blue or yellow, for state 1A/state 2B and state 1B/state 2A, ensuring that the two conditions of a state never had the same ball color or the same direction instruction. The trial sequence of the conditions and the color assignment were counterbalanced over the four days (see Supplementary Table SI) with the first training trial of each of the four states taking place on the first day and the last training trial on the last day. During the baseline session, a non-changing feedback screen with a stationary white ball was shown.

### Instructions

Identical to our previous study (Rance et al., 2014), subjects were told that they could learn to control their own brain activity in previously defined brain regions, the vertical change of the blue or yellow ball being an indicator of the change in brain activation. They were informed that they would be able to observe these changes with a delay of a few seconds and that the two colors represented feedback of different brain regions. It was explained that the goal of the training was to assess if and to what extent it was possible to learn to alter brain activation in these brain areas. The function of the selected brain regions (pain processing) was not mentioned neither was the possibility of a relation between a change in activation to a change in the perception of pain intensity or unpleasantness. Subjects were also unaware that there were only two states and that two conditions per state were identical with regards to feedback computation but were presented in a different ball color. The subjects were advised to not use strategies involving body moment (e.g., muscle tension and relaxation), but were otherwise free to choose any other strategy in the regulation phases. During non-regulation phases, subjects were performing simple mental arithmetic. After each trial the subjects had to rate their perceived control over the ball movement on an 11 point verbal rating scale (ranging from 0 = no control to 10 = absolute control) and the average perceived pain intensity and unpleasantness of the nociceptive stimuli given during the regulation phases. The subjects were asked to report their individual regulation strategies verbally after each training trial.

### rt-fMRI data acquisition and online analysis

The fMRI data were acquired on a 3 T MAGNETOM Trio whole body MR scanner using a standard 12-channel head coil (Siemens Medical Solutions, Erlangen, Germany). Imaging protocols and sequence parameters, as well as the scan procedure and preparation were identical to those used in our previous study (Rance et al., 2014). Before each training day for every subject a standard double gradient-echo field map to measure the static magnetic field was recorded. FMRI data were acquired with a gradient-echo echo planar imaging (EPI) sequence (*TR* = 1500 ms, echo time *TE* = 22 ms, matrix size = 96 × 96, voxel size = 2.2 × 2.2 × 3.5 mm3, gap = 0.5 mm, flip angle α = 90°, bandwidth *BW* = 1270 Hz/px, parallel acquisition technique GRAPPA acceleration factor 2) to record 24 axial slices aligned along the line connecting the anterior-posterior commissure. An anatomical scan was recorded using a three-dimensional (3D) fast low angle shot high-resolution T_1_-weighted anatomical scan (*TR* = 23 ms, *TE* = 5.02 ms, matrix size = 448 × 448, α = 25°, *BW* = 190 Hz/px, voxel size = 0.5 × 0.5 × 1.0 mm^3^) from each subject. To ensure maximum comparability between all scans, all steps of the training, scanning, and feedback procedures were always performed by the same person.

Online data analysis was based on Turbo BrainVoyager Version 1.1 (Brain Innovation, Maastricht, The Netherlands) consisting of online preprocessing including linear detrending, 3D motion detection and correction, as well as drift removal, and computation of statistical maps for every scan based on the General Linear Model (GLM). Feedback computation and visualization was performed with in-house written scripts based on Presentation® Version 13.0 Build 01.23.09 (Neurobehavioral Systems Inc., Albany, CA, USA).

### Offline analyses of the fMRI data

Offline data preprocessing of the fMRI scans was performed using BrainVoyager QX 2.3 (Brain Innovation, Maastricht, The Netherlands). Gradient field map based distortion correction was performed on the EPI images as well as 3D motion correction, spatial smoothing with a Gaussian kernel with a full width half maximum of 8 mm^3^, linear detrending before high frequency artifacts were removed applying a highpass filter (0.006 Hz). Estimation of a response function was done by convolution of a condition box-car time course with a two-gamma hemodynamic response function.

Anatomical datasets were transformed into standard Talairach space and coregistered with their respective functional datasets applying the same transformation parameters using BrainVoyager. A group analysis based on the increase in BOLD percent signal change during the regulation phase with respect to the non-regulation phase was performed using a GLM. All results were Bonferroni corrected for multiple testing.

Offline time course data analysis of the feedback was automated with MATLAB R2011b (The MathWorks Inc., Natick, MA, USA), statistical analyses were performed with IBM SPSS Statistics Desktop (IBM, Armonk, NY, USA) for windows, version 21.0 using 2 (state) × 2 (direction) analysis of variance (ANOVA), paired samples *t*-tests, Pearson correlations, and the Friedman Test for ordinal data. A threshold of *p* < 0.05 was employed to determine statistical significance.

The ROI positions for each trial were equally sized (standard dimension of 12 × 12 × 1 voxels selected on the transverse slice). Time courses for the analyses of the change in the activation difference of the regions (feedback signal) were extracted from the ROI positions that were individually saved on every training trial of each subject for all conditions. For group analyses, the saved ROI positions were averaged per condition and region. For analyses of the activation of single regions requiring correction by an unrelated region to be comparable to the difference feedback, a ROI of equal size was determined after group analyses per condition at a location in Brodman area 39 that did not show activation or deactivation, analogous to our previous study (Rance et al., 2014). Time courses per training trial and subject for each condition were then extracted from this ROI and averaged according to regulation and non-regulation phases for each trial of each condition, leaving six single means per subject and condition for regulation and non-regulation phases. The averaged differences of these six single means were averaged into a resulting mean per subject and condition and trial. Later analyses employed one-tailed significance levels when a specific a priori hypothesis was present and two-tailed significance levels if we had no assumptions about the direction of an association. Group ROI activations for the baseline run and the first and last training trial are shown in Supplementary Tables SII, SIII.

Similarly to Rance et al. (2014) we identified brain regions active upon the painful stimulation alone and those active on the first and last training trial. For all conditions the success and the effect of the regulation training was assessed. We compared the magnitude of the learned regulation and the differential contribution of the single regions and assessed differences in the use of the chosen strategies. The effect of self-regulation (modulation effect) is seen in the BOLD percent signal change of the rACC, the pInsL, as well as the dissociation (difference between rACC and pInsL, constituting the feedback) in a training trial. In the baseline, where no regulation attempts took place, the BOLD percent signal change in the rACC and pInsL as well as their difference reflects the effect of the stimulation. The effect of the training can be seen in the change of the feedback from the first to the last training trial in a condition. Comparing the rACC and pInsL of the first and last training trial can determine whether both regions are similarly affected by the training. Paired samples *t*-tests were used to compare the feedback and single unrelated region corrected signal changes of the rACC and the pInsL of the first and last training trial of every condition to assess the training effect. The baseline stimulation effect was compared to the modulation effect of the first and the last training trial to assess the stability of the activation due to the stimulation as well as changes due to training (see Figure **3** in the Results Section). To achieve state 1A and 1B rACC activation had to be higher than pInsL activation, state 2A and 2B required pInsL activation to be higher than rACC activation. In order to determine, whether successful regulation was possible for all subjects, the modulation effect was calculated for every training trial. To compare states regarding both successful and non-successful regulation attempts and to investigate possible effects of modulation as opposed to effects of non-successful modulation, within each condition subjects were additionally categorized as learners and non-learners. Analogously to our previous study, if the average difference of the activation of the two regions was in the right direction (i.e., the feedback was positive), and this was the case for at least four out of six training trials, and if the modulation effect for the specific condition improved from trial 1 to trial 6, a subject was considered a learner for the condition. The other subjects were categorized as non-learners.

## Results

### Active regions

**Figure 2:**
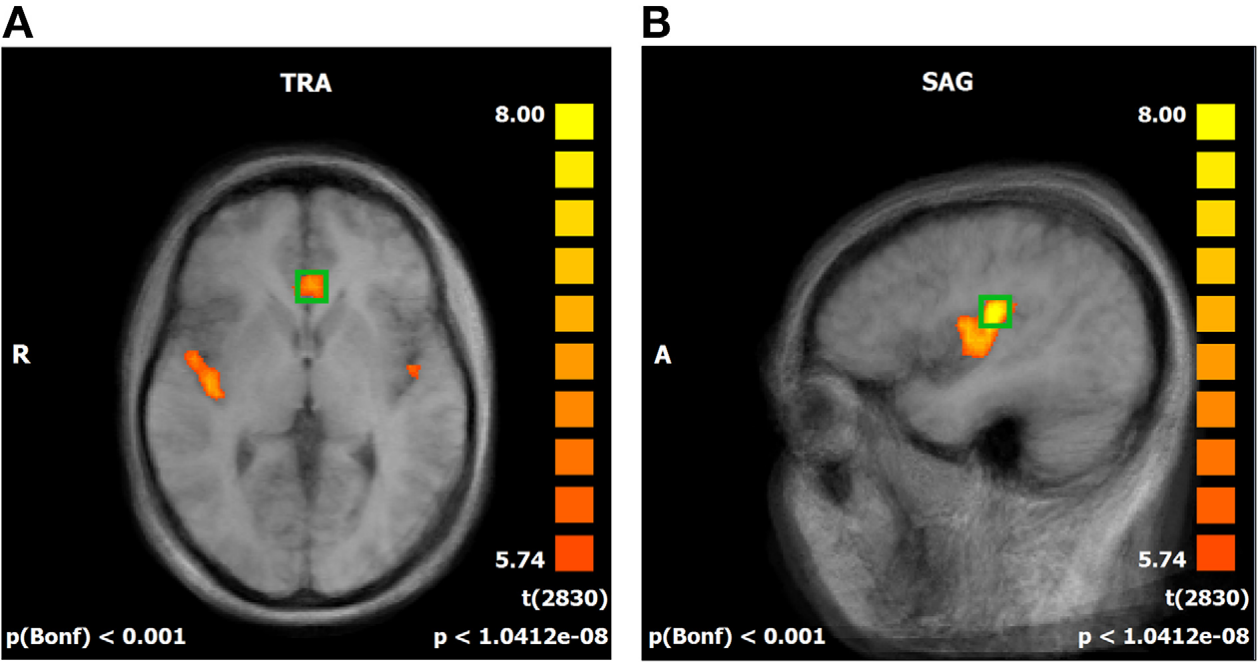
**(A,B)** Offline statistical parametric mapping of the baseline run. Significant activations during the stimulation phases are superimposed over an averaged brain consisting of the ten T_1_-weighted anatomical scans of the subjects, sagittal view (SAG). Red to yellow indicates significant activation in the regulation phases. Individual anatomical and functional scans were transformed into Talairach space. Average activation of the rostral anterior cingulate cortex **(A)** and the left posterior insula **(B)**.

A whole brain random effect analysis of the baseline session where no regulation attempts took place confirmed active clusters suitable for feedback in the rACC and the pInsL (Figure 2). Other active frontal regions were the left and right superior frontal gyrus, the left middle frontal gyrus, left and right inferior frontal gyrus. Parietal regions included the right posterior insula, the secondary somatosensory cortex, the left supramarginal gyrus, and the right precuneus. The left thalamus and caudate nucleus region was also active (see Supplementary Table SII). With the exception of the right posterior insula in the state 1 pInsL—rACC decrease condition (trial 1), all regions found to show either activation or deactivation in the baseline run, were also active on the first training trial and the last training trial. An additional region in the left inferior gyrus was found to be significantly deactivated in all conditions on the first and last training trial but not significantly active in the baseline run. A region in the precuneus was found to be deactivated in the baseline run and activated on the first and last training trial. The right thalamus and caudate nucleus region was found to be active on the first and last training trial in four conditions. For a detailed overview of activated and deactivated regions in the baseline run and the first and last trial of all conditions see Supplementary Tables SII, SIII.

### Training effects

**Figure 3:**
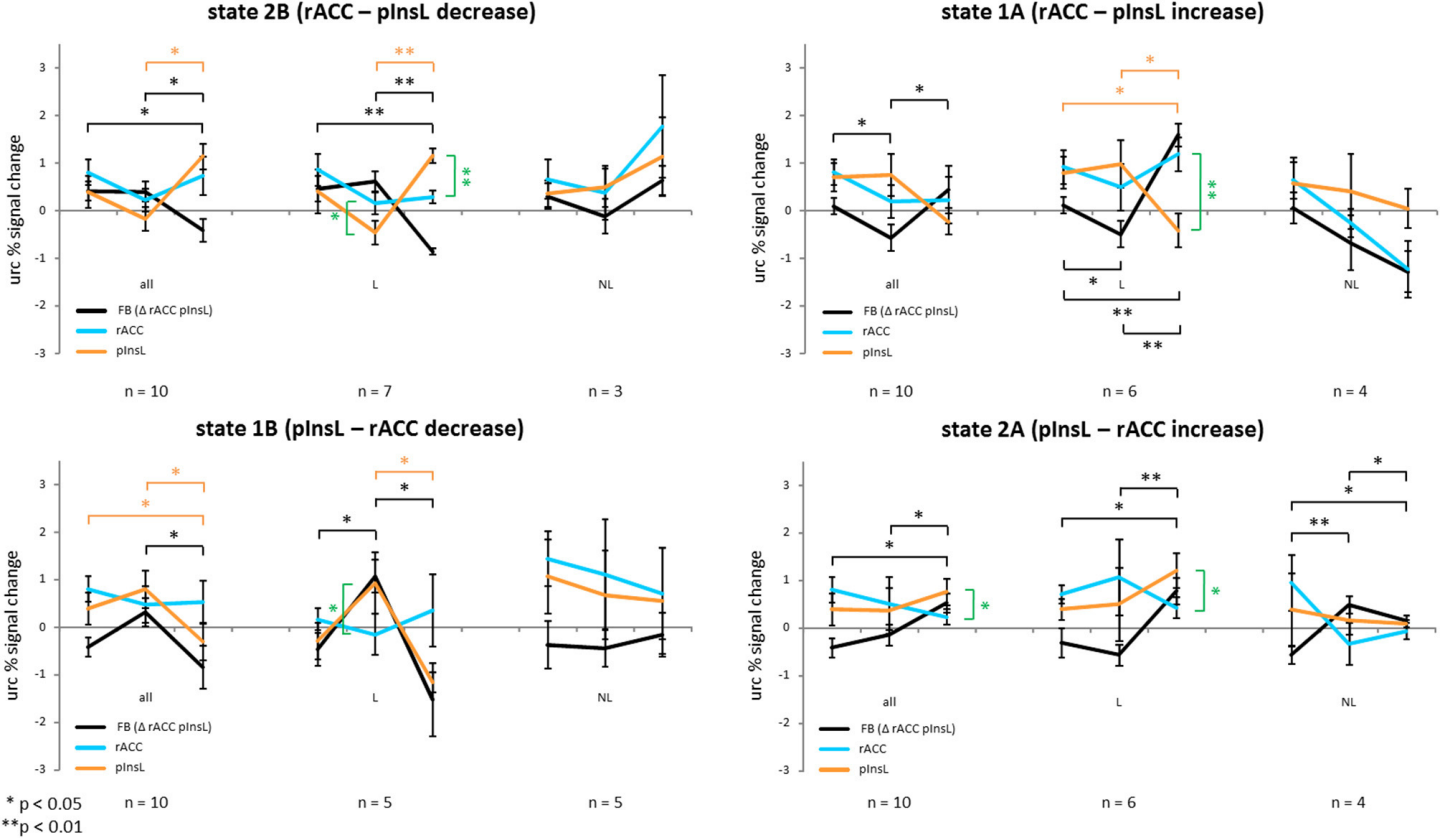
**Unrelated region corrected (urc) percent signal change of all ten subjects of the rostral anterior cingulate cortex (rACC), the left posterior insula (pInsL) and the feedback percent signal change consisting of the activation difference between the regions for the two directions of the two states**. Black lines indicate the change of the feedback signal, red lines the change in the pInsL only, and the blue line the change in the rACC only. Significant changes in the feedback, rACC, and pInsL activation are indicated in brackets of the same color. A significant difference between the activation in the two regions is indicated by a green bracket. The first column depicts the development from the baseline run to the first and the last training trial for all ten subjects, the second for the learners (L) and the third for the non-learners (NL) only. A positive training effect is seen in the significant change of the differences between the regions (feedback) from the first to the last training trial in the right direction.

Subjects trained to achieve two states, each state had two conditions. For state 1 subjects trained to increase the difference between the rACC and pInsL activation with the rACC activation being higher. This state was trained in two conditions, each having a different ball color and an arrow pointing up in one condition and down in the other. State 2 was also trained in two separate conditions, with the goal of increasing the difference between the activation of the regions so that pInsL activation would be higher than rACC activation. The effect of the stimulation is seen in the activation of rACC and pInsL in the baseline. The modulation effect is seen in the activation of the single target regions and the difference of the rACC and pInsL activation of the training trials. This difference was translated into the vertical ball movement, i.e., the feedback signal. The training effect is seen in the magnitude of the difference of the two regions from the first to the last training trial. Figure 3 summarizes the results separately for the four conditions, showing changes in the unrelated region corrected activation of both regions and the difference (feedback signal) for all ten subjects and the subgroups of *learners* and *non-learners*.

#### State 1A (rACC—pInsL increase)

There was a positive training effect with a significantly higher rACC-pInsL difference on the last training trial than on the first [*t*_(9)_ = −1.974; *p* < 0.05] indicating that subjects had learned to significantly increase dissociation between the regions in the indicated direction from the first to the last training trial. The differential brain activation on the first training trial was significantly lower than the difference of the stimulation effects of the regions in the baseline [*t*_(9)_ = 2.544; *p* < 0.05]. The stimulation effect of the rACC and pInsL in the baseline did not differ significantly. The modulation effect of the regions did not differ significantly on the first and the last training trial. There was no significant change in the modulation effect of either the rACC or the pInsL from the first to the last training trial. In the *subgroup of the learners* there was a positive training effect with a significantly higher rACC-pInsL difference on the last training trial than on the first training trial [*t*_(5)_ = −7.995; *p* < 0.001]. The rACC-pInsL difference on the first training trial was significantly lower than the difference of the stimulation effects of the regions in the baseline [*t*_(5)_ = 2.576; *p* < 0.05]. The rACC-pInsL difference on the last training trial [*t*_(5)_ = −8.029; *p* < 0.001] was significantly higher. There was no significant difference in the stimulation effect of the rACC and pInsL and no significant difference between the modulation effects of the regions on the first training trial. We observed a significant difference between the modulation effects of the regions on the last training trial [*t*_(5)_ = 5.950; *p* < 0.01] with the activation in the pInsL being lower. The modulation effect in the pInsL was significantly lower on the last training trial than on the first [*t*_(5)_ = 2.678; *p* < 0.05] and significantly lower than in the baseline [*t*_(5)_ = 3.367; *p* < 0.05]. The modulation effects of the rACC did not change significantly from the first to the last training trial. In the *subgroup of non-learners* there was no significant change modulation effects from the first to the last training trial. There was no significant difference between the stimulation effects of the regions in the baseline and no difference of the modulation effects between the regions on the first and on the last training trial. The modulation effects of the regions did not change from the first to the last training trial.

#### State 1B (pInsL—rACC decrease)

The pInsL-rACC difference on the last training trial was significantly lower than on the first training trial [*t*_(9)_ = 1.860; *p* < 0.05], indicating a positive training effect. The stimulation effect in the baseline and the modulation effect on the first and last training trial of the regions did not differ significantly. There was a significant change in the modulation effect of the pInsL from the first to the last training trial [*t*_(9)_ = 2.340; *p* < 0.05] with the activation being lower on the last training trial. The modulation effect of the pInsL on the last training trial was significantly lower than the stimulation effect in the baseline [*t*_(9)_ = 2.502; *p* < 0.05]. The modulation effect of the rACC did not change significantly from the first to the last training trial. For the *learners* there was a positive training effect with the pInsL-rACC difference on the last training trial being significantly lower than on the first [*t*_(4)_ = 3.198; *p* < 0.05]. The difference of the stimulation effect of the rACC and pInsL in the baseline was significantly lower than the pInsL-rACC difference on the first training trial [*t*_(4)_ = −2.881; *p* < 0.05]. In the baseline there was no significant difference between the stimulation effects of the regions. There was a significant difference between the modulation effects between the regions on the first training trial [*t*_(4)_ = −2.786; *p* < 0.05] with pInsL being lower, but no significant difference between the modulation effects on the last training trial. The modulation effect of the pInsL was significantly lower on the last training trial than on the first training trial [*t*_(4)_ = 3.514; *p* < 0.05]. The modulation effect of the rACC did not change significantly between the trials. In the *subgroup of the non-learners* there was no significant change of the pInsL-rACC difference from the first to the last training trial, the stimulation effect in the baseline did not differ significantly between the regions, nor did the modulation effect differ between the regions on the first and the last training trial.

#### State 2A (pInsL—rACC increase)

There was a positive training effect with the pInsL-rACC difference on the last training trial being significantly higher than on the first training trial [*t*_(9)_ = −2.268; *p* < 0.05]. The modulation effect on the last training trial was significantly higher than the difference of the stimulation effect of the rACC and pInsL in the baseline [*t*_(9)_ = 3.598; *p* < 0.01]. The stimulation effect in the baseline did not differ significantly between the regions, nor did the modulation effect on the first training trial. On the last training trial the modulation effect was significantly higher in the pInsL than in the rACC [*t*_(9)_ = −2.499; *p* < 0.05]. There was no significant change in the modulation effect of the rACC and the pInsL from the first to the last training trial. In the *subgroup of the learners*, the pInsL-rACC difference on the last training trial was significantly higher than on the first [*t*_(5)_ = −7.453; *p* < 0.01] resulting in a positive training effect. The modulation effect on the last training trial was significantly higher than the difference of the stimulation effect of the region in the baseline [*t*_(5)_ = −2.683; *p* < 0.05]. There was no significant difference between the regions' stimulation effect in the baseline. There was a significant difference of the modulation effect between the regions on the last training trial [*t*_(5)_ = 2.69; *p* < 0.05], but no significant change in the modulation effects of either region from the first to the last training trial. For the *non-learners* there was a significant negative training effect with the pInsL-rACC difference in the last training trial being lower than on the first training trial [*t*_(3)_ = 2.649; *p* <0.05]. The difference in the stimulation effect of the regions in the baseline was lower than the pInsL-rACC difference in the first training trial [*t*_(3)_ = −4.788; *p* < 0.05] and the pInsL-rACC difference in the last training trial [*t*_(3)_ = −2.687; *p* < 0.05]. The stimulation effect in the baseline and the modulation effect on the first and last training trials did not differ between the rACC and pInsL. There was no significant change in the modulation effect of the regions from the first to the last training trial.

#### State 2B (rACC – pInsL decrease)

The rACC-pInsL difference on the last training trial was significantly lower than on the first training trial [*t*_(9)_ = 2.024; *p* < 0.05] resulting in a positive training effect, and significantly lower than the difference of the stimulation effect of the regions in the baseline [*t*_(9)_ = 2.214; *p* < 0.05]. There is no significant difference in the stimulation effect of the regions in the baseline and no significant difference between the modulation effects of the regions on the first and last training trial. The modulation effect of the pInsL is significantly higher on the last training trial than on the first [*t*_(9)_ = −3.093; *p* < 0.05]. The modulation effect of the rACC does not change significantly from the first to the last training trial. For the *subgroup of the learners* there was a significant positive training effect with the rACC-pInsL difference being lower on the last than on the first training trial [*t*_(6)_ = 7.861; *p* < 0.001] and significantly lower than the difference of the stimulation effect of the regions in the baseline [*t*_(6)_ = 4.313; *p* < 0.01]. The stimulation effect of the regions did not differ significantly in the baseline. The modulation effect of the regions differed significantly on the first [*t*_(6)_ = 2.792; *p* < 0.05] and on the last training trial [*t*_(6)_ = −11.695; *p* < 0.001]. The modulation effect of the pInsL was significantly higher on the last than on the first training trial [*t*_(6)_ = −6.411; *p* < 0.01], but the modulation effect was not significantly different for the rACC. In the group of the *non-learners*, there was no significant training effect. The stimulation effect in the baseline was not significantly different between the regions, nor was the modulation effect of the regions on the first and last training trial. For an overview of the results see Figure 3.

### Comparison of the controllability of regions

A correlation analysis between the training effects of the conditions showed that there was no correlation between the two conditions of state 1 and state 2. There was a significant negative correlation between the state 1 pInsL—rACC decrease and the state 2 rACC—pInsL decrease conditions [*r*_(8)_ = −0.80; *p* < 0.01].

To directly compare the controllability of the states and conditions, we compared the training effects of the two states and the two directions (increase, decrease). An analysis of variance revealed a significant effect of the regulation direction [*F*_(33.26,0.87)_ = 38.22; *p* < 0.001]. There was no significant effect of the state and no significant interaction effect. Follow-up paired samples *t*-tests between the increase and decrease conditions of the different states, state 1 rACC—pInsL increase and state 2 rACC—pInsL decrease; state 1 pInsL—rACC increase and state 2 pInsL—rACC decrease revealed significant differences in the expected directions in both cases [*t*_(9)_ = 3.14; *p* < 0.05; *t*_(9)_ = 2.48; *p* < 0.05].

**Figure 4:**
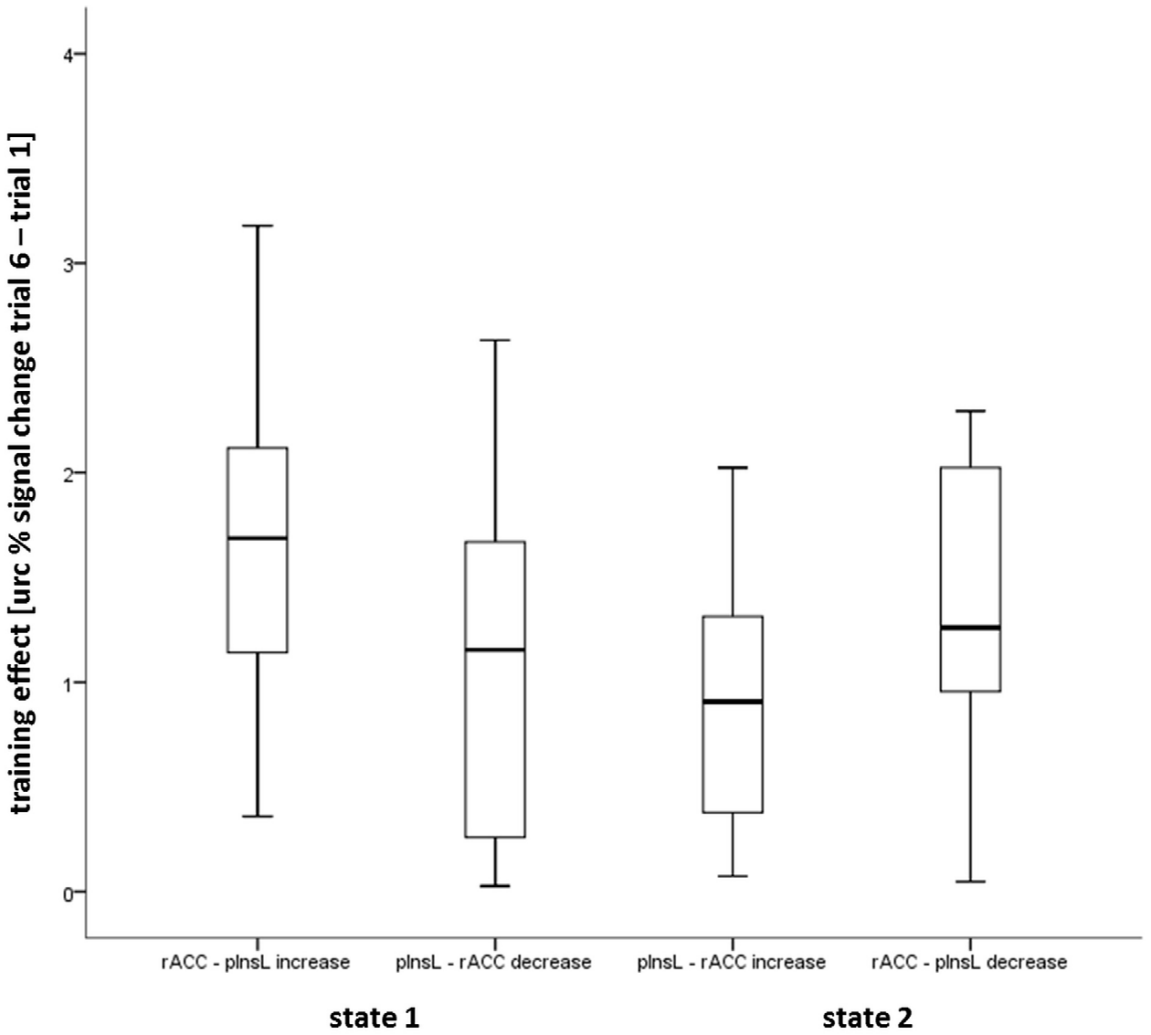
**Box plot showing the median, interquartile range, sample minimum and maximum of the direction independent magnitude of the training effect in the four conditions calculated from the unrelated region corrected (urc) difference of the rostral anterior cingulate gyrus (rACC) and the left posterior insula (pInsL) blood oxygenation level-dependent (BOLD) percent signal change**.

To examine whether one state was easier to achieve, the direction-independent magnitude of the training effect was compared (see Figure 4). There were no significant differences between the four conditions (both states and both directions).

We compared learner and non-learner for each condition. State 1 had six learners in the increase condition and five in the decrease condition, state 2 had six learners in the increase and seven in the decrease condition. Being a learner or a non-learner did not significantly correlate between the four conditions and was not correlated with other person-specific variables such as age, gender, stimulation current, or pain intensity and unpleasantness ratings.

### Effects on pain intensity and unpleasantness ratings

**Figure 5:**
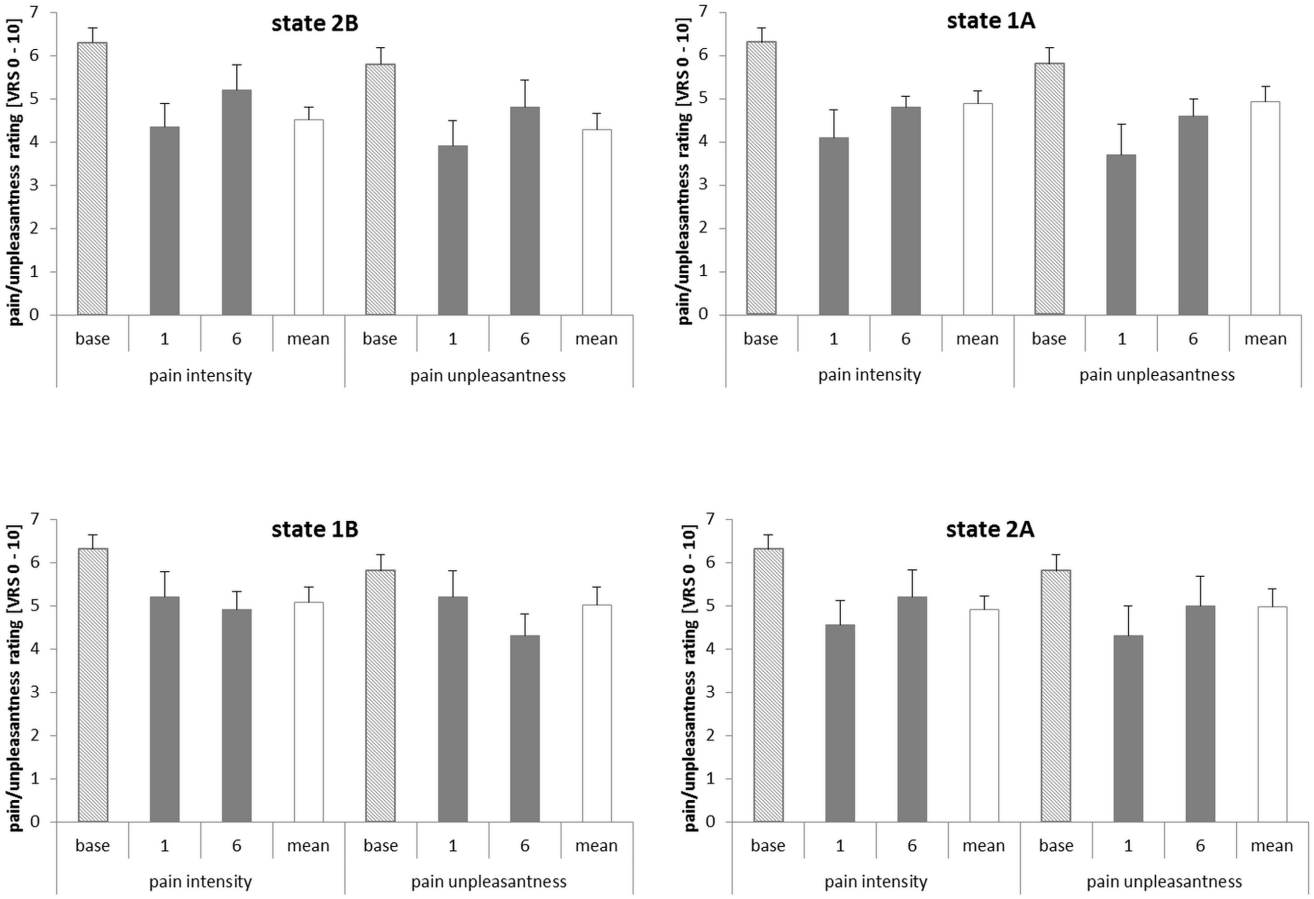
**Pain intensity and unpleasantness ratings in the baseline, on the first and last training trials (trials 1 and 6) and on average over all six training trials**. The pain intensity and pain unpleasantness ratings relate to a verbal rating scale (VRS) with pain intensity ranging from 0 = no pain to 10 = strongest imaginable pain and pain unpleasantness ranging from 0 = not unpleasant to 10 = extremely unpleasant. State 1A, rostral anterior cingulate cortex (rACC) − left posterior insula (pInsL) increase condition; state 1B, pInsL − rACC decrease condition; state 2A, pInsL − rACC increase condition; state 2B, rACC − pInsL decrease condition.

To identify a possible effect of the learned dissociation of the regions on the perception of pain intensity and unpleasantness, paired samples *t*-tests were used to compare the ratings of the first and the last training trial of every condition (see Figure 5). There was no significant difference between the ratings on the first and on the last training trial in any of the four conditions. The same was true for the subgroups of the learners and non-learners. Pain ratings from the baseline run and on the first and on the last training trial did not correlate with rACC and pInsL percent signal change or the difference between the activations.

To investigate the association of the dissociation of rACC and pInsL (training effect) and the evaluation of pain intensity and pain unpleasantness, a correlation analysis was performed. There was no significant correlation between the change in pain intensity and unpleasantness ratings [rating_(training trial 6−1)_] and the respective activation difference between the regions [computed feedback signal_(training trial 6−1)_] in the conditions, neither for the whole group nor for the respective subgroups. A similar correlation analysis between the activation change in the single regions [rACC/pInsL_(training trial 6−1)_] from the first to the last training trial and change in the pain intensity and unpleasantness ratings also revealed no significant correlations.

### Strategies

The reported strategies were subsumed under four categories. Category 1: ball focus: picturing the ball in motion or silent verbal instructions to move the ball; Category 2: distraction: focusing on another body part, continuing with the mental arithmetic, imagining a scene that was not described as being particularly emotional or relaxing; Category 3: positive or negative emotional memories; Category 4: stimulus focus: imagined changing of the stimulus quality or intensity, the location or frequency. If a subject reported to have been using two strategies during one training trial, both were recorded and categorized.

We compared the frequency of the strategy use per category over all conditions using a Friedman test. There was no significant difference between the strategy use over all conditions [χ^2^_(3, 10)_ = 9.255; *p* = 0.51]. For a distribution of the strategies per trial over all conditions see Supplementary Figure S1.

## Discussion

### Active regions during stimulation and modulation

A whole brain offline analysis of the baseline run when subjects received no feedback and did not attempt to modulate brain activation, showed that active regions generally consisted of areas involved in the processing of pain (Apkarian et al., 2005; Iannetti et al., 2005). They included the rACC and pInsL, which were the source of the feedback signal in the following training trials, and other regions that are frequently found to be involved in the processing of pain, including the right posterior insula, bilaterally the secondary somatosensory cortex, and the posterior cingulate cortex in accordance with our previous study (Rance et al., 2014). Additional regions found in the baseline run were the right thalamus and caudate nucleus region, several frontal regions in the mid, inferior, and superior frontal gyrus that showed either activation or deactivation. In the parietal lobe we found deactivation in the left primary somatosensory cortex, the left precuneus, and the left gyrus supramarginalis. Almost all regions found to be active in the baseline were also active on both the first and the last trials of all conditions. One region in the inferior frontal gyrus and the left thalamus and caudate nucleus region was additionally active on the first and last training trials. Similar to our previous study (Rance et al., 2014) the right posterior insula was significantly active in the baseline run, shifting to deactivation on the last training trial in all but one condition. Whereas in the previous study, on the first training trial the pInsR was mainly activated, here the results are mixed, with the pInsR being predominantly deactivated in two conditions of different states, not significantly activated or deactivated in one condition, and activated in one condition. The prevalent change from activation during the painful electric stimulation to deactivation on the last training trial of both regulation of single regions and the difference feedback suggests that this region is not only involved in the processing of pain but also in the regulation effort. Deactivation of this region is observed within the framework of task-induced deactivations seen in the default mode network (Harrison et al., 2011). It was also placed in the pattern of the deactivation of regions involved in the processing of pain during a reduction of pain perception through shifting attention away from the pain or placebo analgesia (Tracey and Mantyh, 2007; Amanzio et al., 2013). An increase in pInsR activation was seen during painful stimulation when subjects were in a state of mindfulness, an increased non-judgmental awareness of present experiences, thus effectively focusing attention on sensory aspects of the stimulus, such as the skin surface of the stimulated area (Gard et al., 2012). Taken together, the results of the present study suggest that during the course of the training the perception of painful stimulation was of increasingly lesser importance, consistent with the increased task demand of the training trials, i.e., finding and memorizing an adequate modulation strategy while observing the feedback and judging the success of the modulation efforts.

A region that was found to be deactivated in the baseline, the right precuneus, was consistently activated in all conditions on the first and the last training trial. The precuneus has been described to be involved in the process of integrating salient stimuli into self-relevant experiences. It is thus not necessarily involved in the processing of pain, but in the evaluation of a salient stimulus in the context of present experience (Cavanna and Trimble, 2006; Goffaux et al., 2014). Increased attention to the stimulation induced feedback might thus lead to an activation of the precuneus independent from the processing of the nociceptive quality of the stimulus.

### Modulation

In our previous study (Rance et al., 2014) the rACC and the pInsL were modulated separately, and subjects learned modulation of all but one condition. Using the same training paradigm in this study with the feedback consisting of a difference feedback of both regions, subjects successfully learned to achieve both states in the two respective conditions. In both studies, there was no significant correlation of the learning success between the conditions and states. When modulation efforts are focused on one region and direction, the absence of a significant correlation of the regulation success between the conditions suggests that subjects are not able to gain control over the activation of one of the regions better than the other and that there is no “easier” direction of modulation. In the present study, subjects trained to increase the difference in the activation of the two regions, with two seemingly different conditions per state. Here, we again did not find evidence that one state is achieved more easily. Moreover, even if one state is successfully learned in one condition, the same state is not necessarily achieved in another condition. We did find a difference in the utilization of the modulated regions. Regarding the regions separately, a significant change from the first to the last training trial was only evident in the pInsL in the state 1 pInsL—rACC decrease and the state 2 rACC—pInsL decrease in the pInsL activation. This was especially true in the subgroup of the learners, where this significant change was additionally observed in the state 1 rACC—pInsL increase condition. These results indicate that the target ROI in the insula was the driving force behind the change in the feedback consisting of the activation difference of the regions. There was also some evidence that the pInsL can be regulated in a more stable manner in our previous study (Rance et al., 2014), where the rACC could be trained overall, however, with a much larger range of training success, especially in the upregulation condition, where the group as a whole did not show a significant training effect. Both the rACC and the pInsL are implicated in a variety of functions. The posterior part of the insula is involved in interoception, emotional processing, and pain perception (Cauda et al., 2012a,b; Dowman, 2014; Uddin et al., 2014). In the presence of ongoing salient painful stimulation, activation in the posterior insula might be more readily accessible. The rACC on the other hand is involved in both emotional and cognitive-evaluative functions apart from playing a crucial role in the network active during pain perception (Bush et al., 2000; Li et al., 2013). It might be involved in both the modulation process itself and the processing of the painful stimulus, yielding it more difficult to regulate especially when the feedback does not depend on rACC activation alone.

Similar to our previous study (Rance et al., 2014), strategy use was equally distributed across the training trials. Together with the result that there also was no significant correlation between learning success between the regions or states, the regulation of rACC and pInsL in the presence of a painful stimulus does not require or favor a specific strategy. This might be connected to the multimodal nature of the regions. In other real-time neurofeedback studies, a concrete strategy has sometimes been linked to the modulation and the function of the modulated area such as emotion induction in the regulation of the right anterior insula (Caria et al., 2007). Because of the distributed nature of pain processing, there might not be generally useful strategies.

### Pain intensity and unpleasantness ratings

In our previous study (Rance et al., 2014) no significant effect of the successful modulation of either the rACC or the pInsL on pain intensity or unpleasantness ratings was found. However, a connection between the modulation in the pInsL and unpleasantness ratings was observed when the difference between the target region (pInsL) and the rACC was high. In the present study, subjects successfully learned to increase the activation difference achieving two different states, however, there was no statistical evidence that the ability to dissociate the two regions was significantly correlated with a change in the pain intensity and unpleasantness ratings. This was also true in the subgroup of learners. Moreover, there was no significant correlation between either the differences of the activation of the target ROIs, or the unrelated region corrected activation of either region, and the pain intensity and unpleasantness ratings. The ratings were not significantly correlated with activation in either region or the combined feedback. Due to the exploratory nature of this study, we examined several specific aspects of feedback modulation and therefor refrained from an overall correction of the *p*-values. Our results are not in line with previous findings by deCharms et al. who found a correlation of the increase and decrease of rACC activation and a change in pain intensity and unpleasantness ratings, thus linking regulation of brain activation with changes in pain perception. One possible reason might be that activation in the rACC and pInsL are the result of both the stimulation or task and modulation efforts (Papageorgiou et al., 2009). In the current literature on neuromodulation there are mixed results on behavioral effects of modulation. Pain perception is a variable experience, involving many modulating cognitive, emotional, and physiological factors (Rhudy and Meagher, 2000; Bantick et al., 2002; Apkarian et al., 2005; Forys and Dahlquist, 2007; Tracey and Mantyh, 2007; Gard et al., 2012) and a wide network of contributing regions (Hofbauer et al., 2001; Tracey and Mantyh, 2007; Iannetti and Mouraux, 2010; Cauda et al., 2012b). The modulation of the perception of pain intensity and unpleasantness of a painful electrical stimulus does not seem possible by regulation of the rACC and pInsL either singularly or combined. Attempting modulation should thus involve the active network to a greater part. This has been suggested especially in the context of rt-fMRI since many connections between brain activation and behavior depend not only on the activity of single regions but on the connectivity of involved networks (Zilverstand et al., 2014). Connectivity feedback seems to be a more feasible method especially when attempting behavioral changes, since changes in connectivity go along with learning to regulate activation in brain areas (Berman et al., 2013; Zotev et al., 2013; Scharnowski et al., 2014). This approach might, moreover, help in identifying regions involved in the process of learning modulation. When considering employing rt-fMRI in the treatment of chronic pain, it is further important to distinguish experimental pain from ongoing chronic pain and the respective activated networks, which may be very different (Baliki et al., 2010). Taken together, rt-fMRI might be valuable in not only identifying regions involved in the processing of pain in pain disorders, but also help understanding the contribution to pain perception of single regions within the active network, which could be the targets of modulation in the treatment of chronic pain.

## Conclusion

In our previous study we found that the state of the network played a crucial role in regulating pain-related activation and might be key in altering the perception of pain. In the present study we therefore explored the extent to which an increase in the difference in the activation of two functionally connected areas in response to a painful stimulus is possible and the effects on the perception of pain. We thus not only examined the results of the group but also looked at differences between subjects who learned regulation and those who did not, similar to the previous study. We found that subjects were able to increase the difference in all four conditions after six trainings trials, thus successfully achieving the two states of either the rACC or the pInsL BOLD percent signal change being higher. When looking at the contribution of the single regions to the combined difference feedback, the pInsL was found to be driving force in three out of the four conditions with significant changes in the activation from the first to the last training trial. Activation in the rACC did not change significantly. In our previous study we saw that control over rACC and pInsL in both directions can be acquired by the majority of subjects (Rance et al., 2014). Mirroring these results, learning success did not correlate between the conditions or states. This indicates that among our subjects, there is no overall ability to learn regulation to achieve the two states in general. This means that subjects who were successful in one or two conditions did not necessarily learn all, and that if a subject learned to establish one state in one condition the same subject did not always learn to establish the state in the other condition. Since the group as a whole did learn to establish both states in the conditions, our results suggest that, given enough training trials, both states can be successfully established. Furthermore, learning was unrelated to chosen strategies. In line with this, the magnitude of the feedback change was similar between all conditions. Despite successful modulation, there was no change in the perception of pain intensity or unpleasantness. Our results suggest that in the modulation of pain intensity and unpleasantness, both the rACC and pInsL either alone or combined, are not sufficient to alter perception of the painful experimental electric stimulus. However, it is possible that increasing automatization of the response would free the respective region from dual tasking and could thus have an effect. This could be tested by using extended training sessions. It is also necessary to identify networks not only involved in the processing of the stimulus but also in learning regulation of the network. It might then be possible to specifically modulate communication of parts of the network to alter perception and even correct altered states of the pain processing network in patients with chronic pain.

### Conflict of interest statement

This study was supported by the Mück-Weymann-Prize of the Deutsche Gesellschaft für Biofeedback e. V. granted to Mariela Rance. The authors declare that the research was conducted in the absence of any commercial or financial relationships that could be construed as a potential conflict of interest.
