# Supplementary material for: Neurofeedback of the difference in activation of the anterior cingulate cortex and posterior insular cortex: two functionally connected areas in the processing of pain
Source: Front Behav Neurosci. 2014 Oct 15;8:357. doi: 10.3389/fnbeh.2014.00357 (PMC4197653; doi:10.3389/fnbeh.2014.00357)
Supplement: Supplementary file 1 [file DataSheet1.DOCX]

|  | day 1 | day 2 | day 3 | day4 |  | day 1 | day 2 | day 3 | day4 |  | day 1 | day 2 | day 3 | day4 |
| --- | --- | --- | --- | --- | --- | --- | --- | --- | --- | --- | --- | --- | --- | --- |
|  |  |  |  |  |  |  |  |  |  |  |  |  |  |  |
| trial | subject 1 | |  |  |  | subject 2 | |  |  |  | subject 3 | |  |  |
| 1 | S1 A | S2 A | S1 B | S2 B |  | S1 B | S1 B | S1 A | S2 B |  | S2 A | S1 A | S1 B | S2 B |
| 2 | S2 B | S1 B | S1 B | S1 A |  | S2 A | S1 B | S1 A | S1 A |  | S1 B | S2 B | S1 B | S2 B |
| 3 | S2 B | S1 B | S2 A | S2 B |  | S1 B | S2 A | S2 B | S2 B |  | S2 A | S2 B | S2 A | S1 A |
| 4 | S1 B | S1 A | S2 B | S1 B |  | S2 B | S2 B | S2 A | S2 A |  | S1 A | S1 B | S1 A | S1 B |
| 5 | S2 A | S1 A | S1 A | S2 A |  | S1 A | S1 A | S2 A | S2 A |  | S1 A | S2 A | S2 B | S2 A |
| 6 | S2 A | S2 B | S1 A | S2 A |  | S1 A | S2 B | S1 B | S1 B |  | S2 B | S1 B | S1 A | S2 A |
|  |  |  |  |  |  |  |  |  |  |  |  |  |  |  |
| trial | subject 4 | |  |  |  | subject 5 | |  |  |  | subject 6 | |  |  |
| 1 | S2 B | S1 B | S2 B | S1 B |  | S1 B | S1 A | S2 A | S2 B |  | S2 A | S1 B | S2 B | S1 A |
| 2 | S1 A | S2 A | S2 B | S1 B |  | S1 B | S2 B | S1 B | S1 A |  | S2 A | S1 B | S1 A | S2 B |
| 3 | S2 B | S2 A | S1 A | S2 A |  | S2 A | S1 A | S2 A | S1 A |  | S1 B | S2 A | S1 A | S2 B |
| 4 | S2 A | S1 A | S2 A | S2 B |  | S2 B | S2 A | S1 A | S2 A |  | S1 A | S2 B | S1 B | S1 B |
| 5 | S1 B | S1 A | S1 B | S1 A |  | S2 B | S1 B | S2 B | S1 B |  | S1 A | S1 A | S1 B | S2 A |
| 6 | S2 A | S2 B | S1 B | S1 A |  | S1 A | S2 A | S2 B | S1 B |  | S2 B | S2 B | S2 A | S2 A |
|  |  |  |  |  |  |  |  |  |  |  |  |  |  |  |
| trial | subject 7 | |  |  |  | subject 8 | |  |  |  | subject 9 | |  |  |
| 1 | S1 A | S1 A | S1 B | S2 A |  | S1 A | S2 A | S1 B | S1 A |  | S1 A | S1 A | S1 B | S2 B |
| 2 | S2 B | S2 B | S1 B | S1 B |  | S2 B | S1 B | S1 B | S2 B |  | S2 B | S2 B | S2 A | S1 A |
| 3 | S2 B | S2 B | S2 A | S1 B |  | S2 B | S1 B | S2 A | S2 B |  | S2 B | S1 A | S2 A | S1 A |
| 4 | S2 A | S2 A | S2 B | S1 A |  | S2 A | S1 A | S2 B | S2 A |  | S2 A | S2 A | S1 A | S1 B |
| 5 | S2 A | S1 B | S1 A | S2 B |  | S2 A | S2 B | S1 A | S1 B |  | S1 B | S1 B | S2 B | S1 B |
| 6 | S1 B | S2 A | S1 A | S1 A |  | S1 B | S1 A | S1 A | S2 A |  | S2 A | S1 B | S2 B | S2 A |
|  |  |  |  |  |  |  |  |  |  |  |  |  |  |  |
| trial | subject 10 | |  |  |  |  |  |  |  |  |  |  |  |  |
| 1 | S1 B | S2 B | S2 A | S1 A |  |  |  |  |  |  |  |  |  |  |
| 2 | S1 B | S1 A | S1 B | S2 B |  |  |  |  |  |  |  |  |  |  |
| 3 | S2 A | S1 A | S2 A | S1 A |  |  |  |  |  |  |  |  |  |  |
| 4 | S2 B | S2 A | S1 A | S2 A |  |  |  |  |  |  |  |  |  |  |
| 5 | S2 B | S1 B | S2 B | S1 B |  |  |  |  |  |  |  |  |  |  |
| 6 | S1 A | S1 B | S2 B | S2 A |  |  |  |  |  |  |  |  |  |  |

Table SI: Listing of the balanced conditions per training day (left to right) and trial (top to bottom) for all ten subjects. Yellow and orange fields indicate a yellow ball color, dark and light blue indicate a blue ball color. S1 A = state 1 rostral anterior cingulate cortex (rACC) - left posterior insula (pInsL) increase condition, S1 B = state 1 pInsL – rACC decrease condition, S2 A = pInsL – rACC increase condition, S2 B = rACC – pInsL decrease condition.

Table SII: Active regions in the baseline. BA = Brodman area, H = Hemisphere, R = right hemisphere, L = left hemisphere, TAL = Talairach coordinates, rACC = rostral anterior cingulate cortex, pInsL = left posterior insula, pInsR = right posterior insula, SI = primary somatosensory cortex, SII = secondary somatosensory cortex, MCC = medial cingulate cortex, SupFroG = superior frontal gyrus, MidFroG = medial frontal gyrus, InFroG = inferior frontal gyrus, supramarg = supramarginal gyrus, PCC = posterior cingulate cortex, Th/Caud = thalamus and caudate nucleus region.

| baseline session | |  | |  | |  | |  | | TAL | |
| --- | --- | --- | --- | --- | --- | --- | --- | --- | --- | --- | --- |
| lobe | H | | structure | Brodman area | *t-*value | | *p-*value | x | y | | z |
|  |  | |  |  |  | |  |  |  | |  |
| limbic |  | | rACC | 24 | 7.355 | | < 0.0001 | -4 | 31 | | 6 |
| parietal | L | | pInsL | 13 | 7.081 | | < 0.0001 | -43 | -5 | | -3 |
| parietal | R | | pInsR | 13 | 8.061 | | < 0.0001 | 41 | -20 | | 14 |
| parietal | L | | SII | 40 | 7.323 | | < 0.0001 | -61 | -29 | | 21 |
| parietal | R | | SII | 40 | 8.369 | | < 0.0001 | 47 | -26 | | 21 |
| limbic |  | | MCC | 24 | 7.682 | | < 0.0001 | -1 | -20 | | 36 |
| frontal | L | | SupFroG | 9 | 9.049 | | < 0.0001 | -16 | 58 | | 30 |
| frontal | R | | SupFroG | 9 | 8.388 | | < 0.0001 | 14 | 58 | | 33 |
| frontal | L | | MidFroG | 6 | 4.910 | | < 0.0001 | -43 | 10 | | 48 |
| limbic | L | | PCC/precuneus | 31 | 5.652 | | < 0.0001 | -13 | -47 | | 30 |
| limbic |  | | MCC | 32 | **-10.369** | | < 0.0001 | -7 | 1 | | 51 |
| frontal | L | | InFroG | 47 | 6.528 | | < 0.0001 | -52 | 31 | | -6 |
| frontal | R | | InFroG | 47 | 5.080 | | < 0.0001 | 44 | 28 | | 0 |
|  | L | | TH/Caud |  | 1.212 | | = 0.226 | -6 | -2 | | 9 |
|  | R | | TH/Caud |  | 2.861 | | = 0.004 | 8 | 3 | | 15 |
| frontal | L | | InFroG | 45 | 3.848 | | < 0.0001 | -58 | 16 | | 12 |
| frontal | R | | InFroG | 45 | 4.872 | | < 0.0001 | 53 | 28 | | 12 |
| parietal | L | | supramarg | 40 | **-13.385** | | < 0.0001 | -37 | -41 | | 39 |
| frontal | L | | InFroG | 9 | **-7.863** | | = 0.326 | -46 | 1 | | 24 |
| parietal | R | | precuneus | 7 | **-0.982** | | < 0.0001 | 23 | -51 | | 54 |
| frontal | L | | MidFroG | 6 | **-10.519** | | < 0.0001 | -25 | -2 | | 51 |
| parietal | L | | SI | 2 | **-11.152** | | < 0.0001 | -41 | -31 | | 33 |
|  | L | | unrel |  | **-1.018** | | = 0.309 | -42 | -61 | | 29 |
|  |  | |  |  |  | |  |  |  | |  |

Table SIII: Activation in the first and last (trial 1 and trial 6) training session for all four conditions. BA = Brodman area, H = Hemisphere, R = right hemisphere, L = left hemisphere, TAL = Talairach coordinates, rACC = rostral anterior cingulate cortex, pInsL = left posterior insula, pInsR = right posterior insula, SI = primary somatosensory cortex, SII = secondary somatosensory cortex, MCC = medial cingulate cortex, SupFroG = superior frontal gyrus, MidFroG = medial frontal gyrus, InFroG = inferior frontal gyrus, supramarg = supramarginal gyrus, PCC = posterior cingulate cortex, Th/Caud = thalamus and caudate nucleus region.

|  |  |  |  | trial 1 |  |  |  |  |  | trial 6 |  |  |  |  |
| --- | --- | --- | --- | --- | --- | --- | --- | --- | --- | --- | --- | --- | --- | --- |
|  |  |  |  |  |  | TAL | | |  |  |  | TAL | | |
| lobe | H | structure | Brodman  area | *t-*value | *p-*value | x | y | z |  | *t-*value | *p-*value | x | y | z |
| State 2  rACC – pInsL  decrease | |  |  |  |  |  |  |  |  |  |  |  |  |  |
| limbic |  | rACC | 24 | 7.045 | < 0.0001 | -7 | 31 | 0 |  | 6.074 | < 0.0001 | 5 | 28 | 15 |
| parietal | L | pInsL | 13 | 5.016 | < 0.0001 | -34 | -8 | -6 |  | 5.747 | < 0.0001 | -39 | 4 | -3 |
| parietal | R | pInsR | 13 | **-3.226** | = 0.001 | 42 | -11 | 14 |  | **-3.177** | = 0.002 | 41 | -8 | 15 |
| parietal | L | SII | 40 | 10.475 | < 0.0001 | -58 | -29 | 23 |  | 8.255 | < 0.0001 | -64 | -32 | 18 |
| parietal | R | SII | 40 | 12.656 | < 0.0001 | 59 | -29 | 27 |  | 11.018 | < 0.0001 | 56 | -29 | 27 |
| limbic |  | MCC | 24 | 6.164 | < 0.0001 | -1 | -17 | 33 |  | 6.271 | < 0.0001 | -4 | -17 | 33 |
| frontal | L | SupFroG | 9 | 11.286 | < 0.0001 | -16 | 49 | 27 |  | 11.573 | < 0.0001 | -16 | 55 | 24 |
| frontal | R | SupFroG | 9 | 7.602 | < 0.0001 | 14 | 55 | 26 |  | 9.733 | < 0.0001 | 14 | 58 | 24 |
| frontal | L | MidFroG | 6 | 10.289 | < 0.0001 | -40 | 16 | 42 |  | 9.709 | < 0.0001 | -40 | 13 | 42 |
| limbic | L | PCC/precuneus | 31 | 7.246 | < 0.0001 | -7 | -44 | 24 |  | 8.637 | < 0.0001 | -10 | -44 | 30 |
| limbic |  | MCC | 32 | **-11.719** | < 0.0001 | -7 | 10 | 45 |  | **-8.389** | < 0.0001 | -7 | 7 | 48 |
| frontal | L | InFroG | 47 | 16.673 | < 0.0001 | -52 | 28 | -6 |  | 14.578 | < 0.0001 | -49 | 31 | -9 |
| frontal | R | InFroG | 47 | 12.740 | < 0.0001 | 44 | 25 | 3 |  | 11.420 | < 0.0001 | 47 | 25 | -5 |
|  | L | TH/Caud |  | 6.811 | < 0.0001 | -4 | 1 | 6 |  | 3.472 | = 0.001 | -7 | 1 | 9 |
|  | R | TH/Caud |  | 7.510 | < 0.0001 | 5 | -2 | 9 |  | 4.087 | < 0.0001 | 5 | 1 | 6 |
| frontal | L | InFroG | 45 | 12.004 | < 0.0001 | -55 | 22 | 9 |  | 8.798 | < 0.0001 | -55 | 19 | 9 |
| frontal | R | InFroG | 45 | 10.947 | < 0.0001 | 56 | 13 | 12 |  | 10.725 | < 0.0001 | 50 | 25 | 3 |
| parietal | L | supramarg | 40 | **-14.345** | < 0.0001 | -37 | -38 | 30 |  | **-13.465** | < 0.0001 | -37 | -41 | 36 |
| frontal | L | InFroG | 9 | **-10.499** | < 0.0001 | -43 | 1 | 24 |  | **-7.930** | < 0.0001 | -43 | 4 | 24 |
| parietal | R | precuneus | 7 | 5.477 | < 0.0001 | 23 | -50 | 54 |  | 6.812 | < 0.0001 | 23 | -50 | 51 |
| frontal | L | MidFroG | 6 | **-12.508** | < 0.0001 | -22 | 4 | 48 |  | **-9.534** | < 0.0001 | -22 | 1 | 45 |
| parietal | L | SI | 2 | **-11.962** | < 0.0001 | -42 | -36 | 34 |  | **-10.715** | < 0.0001 | -43 | -25 | 39 |
|  | L | unrel |  | 1.565 | =0.118 | -50 | -65 | 28 |  | **-1.348** | = 0.178 | -51 | -60 | 27 |
| State 1  rACC – pInsL  increase | |  |  |  |  |  |  |  |  |  |  |  |  |  |
| limbic |  | rACC | 24 | 5.132 | < 0.0001 | -7 | 35 | 9 |  | 4.347 | < 0.0001 | 5 | 32 | 15 |
| parietal | L | pInsL | 13 | 4.435 | < 0.0001 | -34 | -11 | -3 |  | 4.967 | < 0.0001 | -43 | 4 | 0 |
| parietal | R | pInsR | 13 | 4.586 | < 0.0001 | 43 | -31 | 17 |  | 3.090 | = 0.002 | 44 | -2 | 6 |
| parietal | L | SII | 40 | 13.743 | < 0.0001 | -55 | -29 | 21 |  | 7.992 | < 0.0001 | -60 | -26 | 21 |
| parietal | R | SII | 40 | 14.827 | < 0.0001 | 59 | -35 | 30 |  | 11.404 | < 0.0001 | 60 | -29 | 33 |
| limbic |  | MCC | 24 | 6.693 | < 0.0001 | -4 | -20 | 36 |  | 6.097 | < 0.0001 | -7 | -20 | 35 |
| frontal | L | SupFroG | 9 | 11.627 | < 0.0001 | -13 | 61 | 30 |  | 11.136 | < 0.0001 | -17 | 58 | 27 |
| frontal | R | SupFroG | 9 | 9.896 | < 0.0001 | 17 | 61 | 27 |  | 8.344 | < 0.0001 | 12 | 60 | 30 |
| frontal | L | MidFroG | 6 | 10.092 | < 0.0001 | -40 | 16 | 42 |  | 9.881 | < 0.0001 | -40 | 19 | 45 |
| limbic | L | PCC/precuneus | 31 | 4.955 | < 0.0001 | -4 | -47 | 24 |  | 6.772 | < 0.0001 | -10 | -44 | 27 |
| limbic |  | MCC | 32 | **-8.316** | < 0.0001 | -7 | 13 | 45 |  | **-8.888** | < 0.0001 | -7 | 13 | 45 |
| frontal | L | InFroG | 47 | 10.402 | < 0.0001 | -46 | 22 | -15 |  | 6.798 | < 0.0001 | -49 | 31 | -4 |
| frontal | R | InFroG | 47 | 9.838 | < 0.0001 | 54 | 22 | 6 |  | 8.620 | < 0.0001 | 50 | 24 | 0 |
|  | L | TH/Caud |  | 6.463 | < 0.0001 | -7 | 7 | 10 |  | 4.433 | < 0.0001 | -1 | 0 | 5 |
|  | R | TH/Caud |  | 7.610 | < 0.0001 | 5 | 1 | 12 |  | 6.514 | < 0.0001 | 8 | -2 | 11 |
| frontal | L | InFroG | 45 | 9.652 | < 0.0001 | -55 | 19 | 12 |  | 7.598 | < 0.0001 | -58 | 19 | 12 |
| frontal | R | InFroG | 45 | 14.075 | < 0.0001 | 56 | 19 | 18 |  | 9.902 | < 0.0001 | 56 | 19 | 18 |
| parietal | L | supramarg | 40 | **-8.492** | < 0.0001 | -37 | -38 | 33 |  | **-11.908** | = 0.0100 | -40 | -38 | 36 |
| frontal | L | InFroG | 9 | **-11.920** | < 0.0001 | -43 | 1 | 24 |  | **-5.549** | < 0.0001 | -42 | 1 | 22 |
| parietal | R | precuneus | 7 | 7.286 | < 0.0001 | 23 | -50 | 51 |  | 9.726 | < 0.0001 | 20 | -53 | 51 |
| frontal | L | MidFroG | 6 | **-9.019** | < 0.0001 | -22 | 1 | 51 |  | **-11.156** | < 0.0001 | -25 | 4 | 48 |
| parietal | L | SI | 2 | **-8.640** | < 0.0001 | -46 | -29 | 39 |  | **-11.070** | < 0.0001 | -43 | -27 | 42 |
|  | L | unrel |  | **1.092** | = 0.275 | -41 | -60 | 25 |  | **-0.889** | =0.374 | -43 | -65 | 33 |
| State 1  pInsL – rACC  decrease | |  |  |  |  |  |  |  |  |  |  |  |  |  |
| limbic |  | rACC | 24 | 5.774 | < 0.0001 | -10 | 31 | 9 |  | 6.678 | < 0.0001 | -4 | 31 | 17 |
| parietal | L | pInsL | 13 | 2.643 | = 0.008 | -36 | -8 | -6 |  | 5.434 | < 0.0001 | -46 | -2 | -5 |
| parietal | R | pInsR | 13 | 1.732 | = 0.083 | 34 | -14 | 13 |  | **-5.600** | < 0.0001 | 41 | -11 | 15 |
| parietal | L | SII | 40 | 8.299 | < 0.0001 | -55 | -32 | 21 |  | 9.237 | < 0.0001 | -58 | -35 | 12 |
| parietal | R | SII | 40 | 8.870 | < 0.0001 | 62 | -23 | 24 |  | 11.498 | < 0.0001 | 62 | -38 | 27 |
| limbic |  | MCC | 24 | 3.672 | < 0.0001 | 5 | -20 | 28 |  | 8.049 | < 0.0001 | -1 | -20 | 33 |
| frontal | L | SupFroG | 9 | 7.428 | < 0.0001 | -16 | 49 | 30 |  | 12.116 | < 0.0001 | -16 | 55 | 30 |
| frontal | R | SupFroG | 9 | 5.618 | < 0.0001 | 14 | 66 | 24 |  | 10.679 | < 0.0001 | 14 | 52 | 27 |
| frontal | L | MidFroG | 6 | 7.098 | < 0.0001 | -40 | 13 | 45 |  | 10.281 | < 0.0001 | -40 | 13 | 42 |
| limbic | L | PCC/precuneus | 31 | 6.877 | < 0.0001 | -7 | -44 | 30 |  | 8.274 | < 0.0001 | -13 | -41 | 30 |
| limbic |  | MCC | 32 | **-10.534** | < 0.0001 | -7 | 10 | 45 |  | **-12.401** | < 0.0001 | -10 | 10 | 42 |
| frontal | L | InFroG | 47 | 10.679 | < 0.0001 | -46 | 31 | -3 |  | 12.552 | < 0.0001 | -49 | 31 | -12 |
| frontal | R | InFroG | 47 | 9.638 | < 0.0001 | 47 | 28 | -3 |  | 10.831 | < 0.0001 | 44 | 25 | -9 |
|  | L | TH/Caud |  | 7.243 | < 0.0001 | -7 | 10 | 6 |  | 2.362 | = 0.018 | -7 | 1 | 8 |
|  | R | TH/Caud |  | 6.965 | < 0.0001 | 8 | 7 | 9 |  | 3.826 | < 0.0001 | 8 | -8 | 15 |
| frontal | L | InFroG | 45 | 9.218 | < 0.0001 | -49 | 25 | 0 |  | 6.960 | < 0.0001 | -49 | 24 | 3 |
| frontal | R | InFroG | 45 | 9.539 | < 0.0001 | 56 | 19 | 3 |  | 10.653 | < 0.0001 | 56 | 22 | 18 |
| parietal | L | supramarg | 40 | **-11.057** | < 0.0001 | -37 | -35 | 33 |  | **-15.187** | < 0.0001 | -40 | -41 | 33 |
| frontal | L | InFroG | 9 | **-5.978** | < 0.0001 | -46 | 7 | 27 |  | **-12.093** | < 0.0001 | -46 | 4 | 24 |
| parietal | R | precuneus | 7 | 5.292 | < 0.0001 | 20 | -53 | 52 |  | 2.652 | = 0.008 | 20 | -53 | 55 |
| frontal | L | MidFroG | 6 | **-7.112** | < 0.0001 | -22 | 4 | 48 |  | **-13.732** | < 0.0001 | -22 | 1 | 45 |
| parietal | L | SI | 2 | **-10.301** | < 0.0001 | -40 | -30 | 36 |  | **-10.507** | < 0.0001 | -46 | -29 | 39 |
|  | L | unrel |  | **0.607** | =0.544 | -45 | -60 | 27 |  | **-1.309** | =0.191 | -45 | -58 | 30 |
| State 2  pInsL – rACC  increase | |  |  |  |  |  |  |  |  |  |  |  |  |  |
| limbic |  | rACC | 24 | 3.395 | < 0.0001 | -5 | 24 | 0 |  | 5.038 | < 0.0001 | -4 | 35 | 2 |
| parietal | L | pInsL | 13 | 3.060 | < 0.0001 | -37 | -11 | -3 |  | 3.117 | = 0.002 | -34 | -2 | -9 |
| parietal | R | pInsR | 13 | 3.246 | < 0.0001 | 44 | -20 | 14 |  | 3.596 | < 0.0001 | 44 | -20 | 24 |
| parietal | L | SII | 40 | 13.793 | < 0.0001 | -55 | -36 | 27 |  | 9.693 | < 0.0001 | -58 | -38 | 23 |
| parietal | R | SII | 40 | 11.907 | < 0.0001 | 59 | -20 | 30 |  | 10.982 | < 0.0001 | 53 | -29 | 27 |
| limbic |  | MCC | 24 | 6.012 | < 0.0001 | -10 | -17 | 36 |  | 6.471 | < 0.0001 | -1 | -17 | 36 |
| frontal | L | SupFroG | 9 | 12.580 | < 0.0001 | -16 | 58 | 30 |  | 13.804 | < 0.0001 | -16 | 58 | 27 |
| frontal | R | SupFroG | 9 | 12.133 | < 0.0001 | 14 | 61 | 33 |  | 11.006 | < 0.0001 | 14 | 58 | 27 |
| frontal | L | MidFroG | 6 | 10.627 | < 0.0001 | -40 | 19 | 47 |  | 7.890 | < 0.0001 | -40 | 22 | 48 |
| limbic | L | PCC/precuneus | 31 | 7.964 | < 0.0001 | -13 | -41 | 30 |  | 10.370 | < 0.0001 | -10 | -44 | 30 |
| limbic |  | MCC | 32 | **-10.560** | < 0.0001 | -7 | 13 | 42 |  | **-13.112** | < 0.0001 | -7 | 7 | 48 |
| frontal | L | InFroG | 47 | 8.473 | < 0.0001 | -46 | 34 | -6 |  | 9.335 | < 0.0001 | -49 | 28 | -12 |
| frontal | R | InFroG | 47 | 10.608 | < 0.0001 | 47 | 25 | -3 |  | 8.068 | < 0.0001 | 47 | 28 | -3 |
|  | L | TH/Caud |  | 4.499 | < 0.0001 | -7 | 4 | 9 |  | 4.114 | < 0.0001 | -7 | -2 | 0 |
|  | R | TH/Caud |  | 5.284 | < 0.0001 | 5 | -2 | 9 |  | 4.101 | < 0.0001 | 8 | 7 | 10 |
| frontal | L | InFroG | 45 | 8.430 | < 0.0001 | -52 | 25 | 6 |  | 8.191 | < 0.0001 | -55 | 22 | 3 |
| frontal | R | InFroG | 45 | 11.597 | < 0.0001 | 56 | 13 | 15 |  | 7.371 | < 0.0001 | 47 | 25 | 3 |
| parietal | L | supramarg | 40 | **-10.318** | < 0.0001 | -37 | -41 | 33 |  | **-14.539** | < 0.0001 | -40 | -38 | 33 |
| frontal | L | InFroG | 9 | **-6.964** | < 0.0001 | -43 | 7 | 24 |  | **-10.132** | < 0.0001 | -46 | 4 | 24 |
| parietal | R | precuneus | 7 | 12.090 | < 0.0001 | 17 | -53 | 53 |  | 6.407 | < 0.0001 | 20 | -50 | 51 |
| frontal | L | MidFroG | 6 | **-5.914** | < 0.0001 | -22 | 4 | 48 |  | **-13.147** | < 0.0001 | -25 | 1 | 45 |
| parietal | L | SI | 2 | **-7.895** | < 0.0001 | -43 | -31 | 36 |  | **-11.620** | < 0.0001 | -43 | -30 | 33 |
|  | L | unrel |  | 0.923 | =0.356 | -43 | -65 | 35 |  | **-0.873** | =0.383 | -43 | -65 | 38 |
|  |  |  |  |  |  |  |  |  |  |  |  |  |  |  |
|  |  |  |  |  |  |  |  |  |  |  |  |  |  |  |


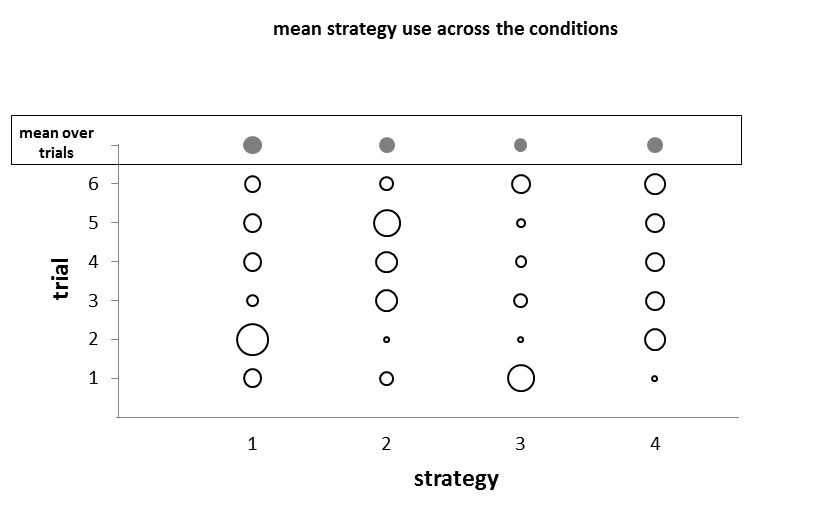


Figure S1: Distribution of strategy use in all six trials for the four conditions (white) and the mean strategy use across the six trials for the conditions (gray). The size of the circles corresponds to the frequency a strategy is used by the subjects. The strategy use did not differ between the conditions in any trial, nor did the mean strategy use. There were no significant differences in strategy use for any condition across the trials.
